# Vitamin D resistance in chronic kidney disease (CKD)

**DOI:** 10.1186/1471-2369-15-47

**Published:** 2014-03-19

**Authors:** Amay Parikh, Herbert S Chase, Linda Vernocchi, Leonard Stern

**Affiliations:** 1 Nephrology, Columbia University, New York, NY, USA; 2 Biomedical Informatics, Columbia University, New York, NY, USA

**Keywords:** Cholecalciferol, Vitamin D deficiency, Parathyroid hormone, Chronic kidney disease, Kidney transplant

## Abstract

**Background:**

Previous studies have shown that treatment with ergocalciferol in patients with CKD stage 3 + 4 is not effective with less than 33% of patients achieving a 25-OH vitamin D target of >30 ng/ml. The aim of this study was to test the response to cholecalciferol in CKD. We attempted to replete 25-OH vitamin D to a target level of 40–60 ng/ml using the response to treatment and PTH suppression as an outcome measure.

**Methods:**

This retrospective cohort study identified patients (Stages 2–5 and Transplant) from 2001–2010 who registered at the Chronic Kidney Disease Clinic. Patients received cholecalciferol 10,000 IU capsules weekly as initial therapy. When levels above 40 ng/ml were not achieved, doses were titrated up to a maximum of 50,000 IU weekly. Active vitamin D analogs were also used in some Stage 4–5 CKD patients per practice guidelines. Patients reaching at least one level of 40 ng/mL were designated RESPONDER, and if no level above 40 ng/mL they were designated NON-RESPONDER. Patients were followed for at least 6 months and up to 5 years.

**Results:**

352 patients were included with a mean follow up of 2.4 years. Of the CKD patients, the initial 25-OH vitamin D in the NON-RESPONDER group was lower than the RESPONDER group (18 vs. 23 ng/ml) (p = 0.03). Among all patients, the initial eGFR in the RESPONDER group was significantly higher than the NON-RESPONDER group (36 vs. 30 ml/min/1.73 m^2^) (p < 0.001). Over time, the eGFR of the RESPONDER group stabilized or increased (p < 0.001). Over time, the eGFR in the NON-RESPONDER group decreased toward a trajectory of ESRD. Proteinuria did not impact the response to 25-OH vitamin D replacement therapy. There were no identifiable variables associated with the response or lack of response to cholecalciferol treatment.

**Conclusions:**

CKD patients treated with cholecalciferol experience treatment resistance in raising vitamin D levels to a pre-selected target level. The mechanism of vitamin D resistance remains unknown and is associated with progressive loss of eGFR. Proteinuria modifies but does not account for the vitamin D resistance.

## Background

Vitamin D [25(OH)D] insufficiency and secondary hyperparathyroidism is widely prevalent in patients with chronic kidney disease [1] including patients who have received a renal transplant [2]. The Kidney Disease Outcomes Quality Initiative (K/DOQI) guidelines recommend measuring PTH and initiating treatment of vitamin D insufficiency starting with CKD stage 3 [3].

Dietary intake of vitamin D may originate from either plant based sources (ergocalciferol, D2) or animal based sources (cholecalciferol, D3). The metabolic pathways of activating vitamin D2 or D3 are essentially the same. Active vitamin D2 and D3 both target the vitamin D receptor initiating vitamin D regulated gene transcription [4]. Achieving optimal levels of serum 25(OH) D remains a challenge in patients with advanced chronic kidney disease. Not only is 25-OH vitamin D purported to have its own beneficial effects, but metabolism to calcitriol has been shown to reduce PTH levels [1,5]. The National Kidney Foundation (NKF) guidelines state that optimal 25(OH) D levels should be greater than 30 ng/mL and should be repleted with oral ergocalciferol [3] while the American Journal of Nutrition guidelines recommend levels greater than 40 ng/mL [6,7]. There are no specific recommendations in the NKF/KDOQI guidelines regarding vitamin D insufficiency for kidney transplant recipients or patients receiving dialysis. There is not much data available to suggest exactly how much ergocalciferol should be administered [8-10] and whether such therapy impacts vitamin D and serum PTH concentrations. Zissman et al. studied the response to ergocalciferol for CKD Stages 3 and 4 [11]. They found a partial reduction in PTH for patients with Stage 3 CKD but not Stage 4 when repleted with ergocalciferol. Less than 33% of patients achieved a 25-vitamin D (Vit D) target of >30 ng/ml and 50% did not respond to treatment [11]. Al-Aly et al. found similar findings and noted a similar lack of response to ergocalciferol [5].

Cholecalciferol (D3) is presumably more potent than ergocalciferol (D2) [4,12,13]. The aim of this study was to test the response to cholecalciferol in CKD. Similar to that observed for ergocalciferol, suggested dosages for repletion vary [14,15]. We attempted to replete 25-OH vitamin D levels to a target of 40–60 ng/mL using the response to treatment and PTH suppression as outcome measures. The working hypothesis for this study was that patients with more advanced CKD require significantly higher dosages to achieve the pre-selected blood level target. Patients with better kidney function would respond more readily to vitamin D supplementation compared to those with poorer function.

## Methods

### Patients

570 patients were identified from 2001 to 2010 who registered at the Chronic Kidney Disease (CKD) Clinic at Columbia University Medical Center (CUMC). Patient data measurements were electronically extracted from the clinical data warehouse (CDW), the research database of the Columbia University Medical Center (CUMC) of the New York Presbyterian Hospital (NYPH) system. Data extracted from this cohort included demographic information, serum creatinine, 25-OH and 1,25-OH vitamin D measurements, calcium, albumin, parathyroid hormone levels, proteinuria levels (24 hr. urine protein or protein/creatinine ratio) and other laboratory tests. Diabetes and hypertension status was assigned from ICD-9 coding.

### Study design

Patients received vitamin D repletion with cholecalciferol 10,000 IU capsules weekly. Patients also received dietary counseling to reduce daily calcium intake to less than 500 mg and daily phosphate intake to less than 1000 mg. At each patient follow-up visit, the medication list was reviewed and compliance was confirmed. When levels of 25-OH vitamin D above 40 ng/mL were not achieved, doses were titrated upwards over 6 months to a maximum of 50,000 IU weekly. A step-wise protocol was followed to increase the dosage of cholecalciferol. Follow-up, repeat vitamin D levels, and dose adjustment as needed was performed at regular intervals concurrent with clinic visits at 1–4 month intervals. 25-OH vitamin D levels were measured by a chemiluminescent immunoassay (ARUP Laboratories; Salt Lake City, UT). The same laboratory and assay were used throughout the study period.

Responsiveness, as measured by the change in 25-OH vitamin D level, was the primary outcome measure. PTH suppression was a secondary outcome measure. Safety markers included hypercalcemia and hyperphosphatemia. eGFR was calculated for patients using the 4 variable modified diet in renal disease (MDRD) equation and extracted creatinine and demographic values [16].

### Study population

Patients who maintained 25-OH vitamin D levels ≥40 ng/mL and never fell below this level were designated REPLETE. When a patient’s 25-OH vitamin D level fell below 40 ng/mL, the patient was followed until a response was noted (i.e. 25-OH vitamin D level ≥ 40 ng/mL). A response was defined as achieving a level of at least 40 ng/mL in a period no less than 6 months. Patients achieving vitamin D levels of ≥40 ng/ml with cholecalciferol were designated as RESPONDER. If the vitamin D level did not reach 40, the patient was designated NON-RESPONDER. Patients were excluded who had only one measurement of 25-OH vitamin D during the study period, or who had no follow-up beyond 6 months.

### Statistical analysis

Chi-squared or Fisher’s exact test was used to compare categorical variables; t-test or Wilcoxon Rank Sum test was used to compare continuous variables. Significance was defined at a 95% confidence level for two-tail measurement. Longitudinal effects of 25-OH vitamin D were analyzed by a linear mixed effect model with random intercept, which accounted for individual differences in the initial outcome value. Analysis was performed to adjust for varying follow-up times. A time adjusted variable was introduced to reduce observer bias which decreased the N. Repeated measures ANOVA was not utilized due to missing values, unequal time differences between data points, and the inability to adjust for a number of covariates.

Statistical analysis was performed using SAS version 9.2 (SAS Institute Inc., Cary, NC). Columbia University Medical Center Institutional Review Board, FWA# 00002636, approved this protocol (IRB Protocol # AAAD8498) in compliance with the Helsinki Declaration from 8/6/09 to present. There was no interaction or intervention with any human subjects. This data is not publicly available. The IRB granted a waiver of consent for this retrospective cohort study.

## Results

**Figure 1:**
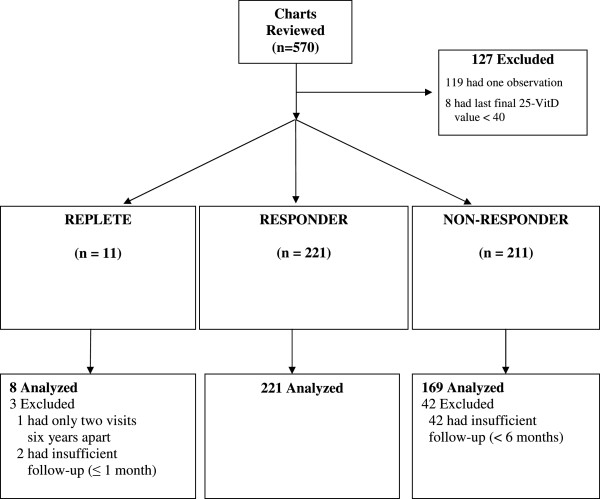
Study flow diagram.

Of the 570 patients identified from 2000 to 2010 who were treated at the Chronic Kidney Disease Clinic, 127 were initially excluded (Figure 1). 8 patients were analyzed who consistently maintained 25-OH vitamin D levels > 40 ng/mL. These patients were designated REPLETE. Over the 10 year period, the 25-OH vitamin D level fell below 40 ng/mL in 221 patients, and was successfully repleted with cholecalciferol supplementation. These patients were designated RESPONDER. Supplementation with cholecalciferol failed to achieve a level of 40 ng/ml or greater in 169 patients who were designated NON-RESPONDER.

Table 1 shows the distribution of patients between 3 groups: CKD, post-transplant (TXP), and renal replacement therapy (RRT). Statistically significant differences between groups were observed in age (p < 0.001) and for hypertensive renal disease (p = 0.03).

**Table 1:** Demographics

	**CKD**	**Transplant**	**RRT**	**P value**^**‡**^
N	309	43	46	
Males	162 (52.4%)	24 (55.8%)	25 (54.4%)	0.90
Age^†^	66·4 (14.2)	51·6 (11.9)	58.1 (17.5)	<0.001
Diabetes, only	1 (0.3%)	0 (0·0%)	0 (0.0%)	1.0
Hypertension, only	123 (39.8%)	25 (58.1%)	15 (32.6%)	0.03
Diabetic and hypertensive	171 (55.3%)	18 (41.9%)	29 (63.0%)	0.12
Non-diabetic and non-hypertensive	14 (4.5%)	0 (0.0%)	2 (4.4%)	0.44

^†^Categorical variables are expressed as count (%); age as mean (SD); all other continuous variables as median (25^th^ percentile-75^th^ percentile).

^‡^Based on chi-squared or Fisher’s exact test for categorical variables; t-test or Wilcoxon Rank Sum test for continuous variables.

Table 2 shows the distribution of patients by vitamin D status. Of the 309 CKD patients, 54.7% of CKD patients are RESPONDER compared to 42.7% NON-RESPONDER (p < 0.001). 81.4% of the 43 TXP patients were RESPONDER compared to 18.6% NON-RESPONDER. Among all patients, unadjusted initial values for eGFR, Albumin, Phosphate, and 1, 25-OH vitamin D were significantly different between the RESPONDER and NON-RESPONDER groups. In the patients with CKD stages 3–5, the unadjusted initial mean eGFR and 25-OH vitamin D were lower in the NON-RESPONDER compared to the RESPONDER (p < 0.03 and p < 0.001, respectively). Table 3 shows the initial values of the subset of patients for whom proteinuria data was available. Significant differences in initial mean 25-OH vitamin D (p = 0.02) and mean proteinuria (p < 0.004) were noted.

**Table 2:** Primary and secondary outcomes

	**Non-responder**	**Responder**	**Replete**	**P Value**^**‡ **^**(responder vs. non-responders)**
N	169	221	8	
Males	86 (50.9%)	119 (53.9%)	6 (75.0%)	0.56
Age^†^	62.5 (15·5)	64.9 (14·9)	64.5 (18·0)	0.12
Diabetes, only	0 (0.0%)	1 (0.45%)	0 (0.0%)	1.0
Hypertension, only	62 (36.7%)	97 (43.9%)	4 (50.0%)	0.15
Diabetic and hypertensive	102 (60.4%)	113 (51.1%)	3 (37.5%)	0.07
Non-diabetic and non-hypertensive	5 (3.0%)	10 (4.5%)	1 (12.5%)	0.43
CKD	132 (78.1%)	169 (76.5%)	8 (100.0%)	**<0.001**
Transplant	8 (4.7%)	35 (15.8%)	0 (0.0%)	
RRT	29 (17.2%)	17 (7.7%)	0 (0.0%)	
eGFR	30.0 (22.0)	36·0 (27.0)		**<0.001**
PTH	99.0 (137.0)	86·5(102.0)		0.08
Albumin	3.9 (0.6)	4·3 (0.4)		**<0.001**
Initial mean phosphate	3.8 (1.1)	3·5 (0.9)		**<0.001**
Initial mean 1,25-OH vitamin D	20.5 (18.0)	30.0 (19.0)		**<0.001**
For CKD stages 3–5 only				
Initial mean eGFR	28·3 (11.5)	31·5 (11.2)		**0.0253**
Initial mean 25-OH vitamin D	18·3 (9.0)	23·6 (9.1)		**<0.001**

^†^Categorical variables are expressed as count (%); age as mean (SD); all other continuous variables as median (25^th^ percentile-75^th^ percentile).

^‡^Based on chi-squared or Fisher’s exact test for categorical variables; t-test or Wilcoxon Rank Sum test for continuous variables.

^†^At time of 25-vitamin D grouping.

^‡^Prior to 25-vitamin D grouping.

The boldface items indicate values less than 0.05.

**Table 3:** Outcomes by group

	**Non-responder**	**Responder**	**P value**
*Initial Mean 25VitD	19 ng/mL	23 ng/mL	**0.02**
*Initial Mean Proteinuria	1.47 g/day	0.89 g/day	**<0.004**
*Initial Mean eGFR (ml/min/1·73 m^2^)	27	31	0.09
*Initial PTH	110 pg/mL	100 pg/mL	0.38

*Not adjusted for follow-up time. Closely concurrent lab values were measured with initial 25-OH Vitamin D.

The boldface items indicate values less than 0.05.

**Figure 2:**
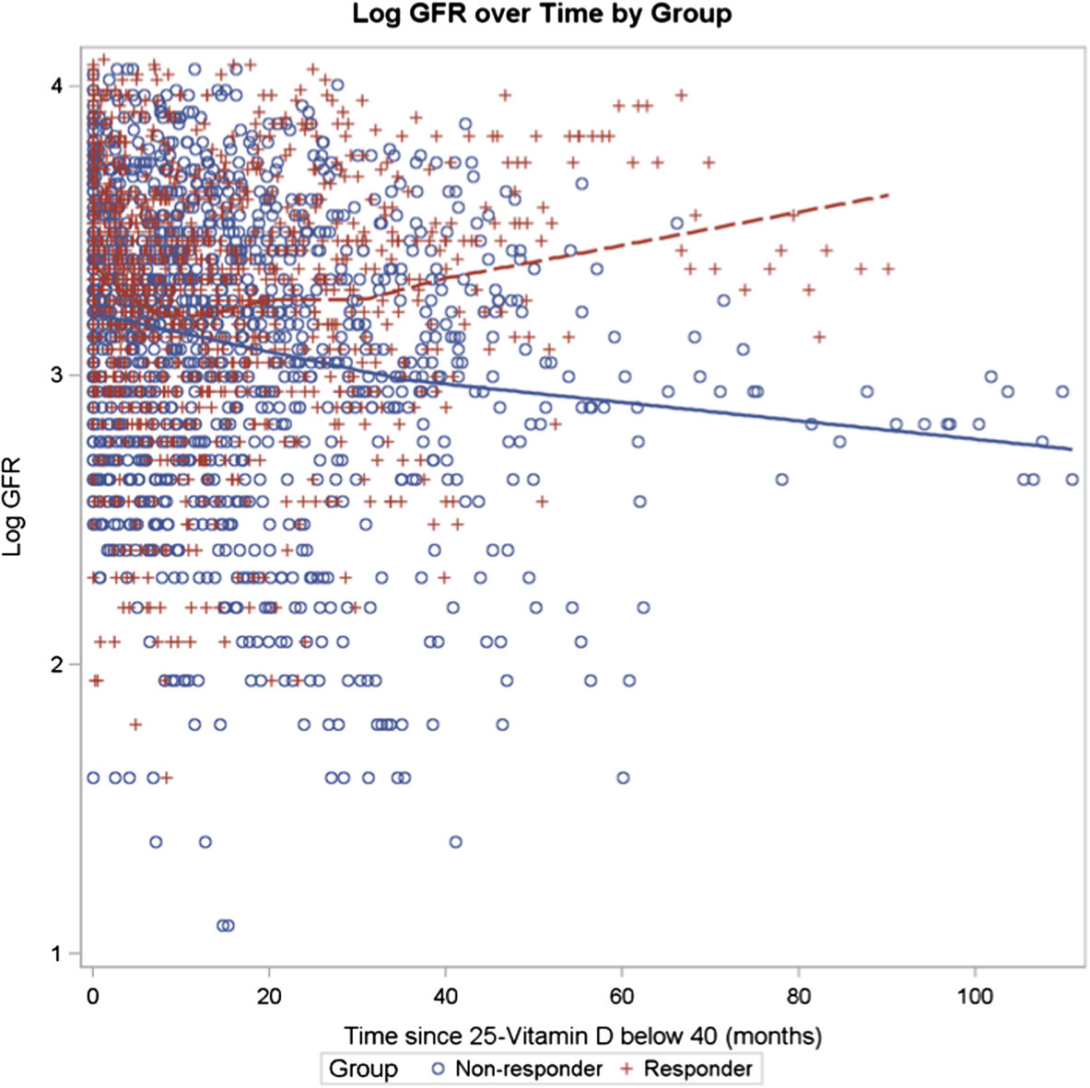
**Log eGFR over time by group for CKD Stages 3–5.** Log eGFR over Time by Group for CKD patients with eGFR < 60 ml/min/1.73 m^2^. N = 108 Non-responders and 132 Responders. No differences in the distribution of CKD stages between the two groups were noted (p = 0.21).

Figure 2 depicts the relationship between log eGFR versus time in the RESPONDER and NON-RESPONDER groups for patients where the 25-OH vitamin D was below 40 ng/mL for CKD patients Stage 3–5 (N = 240) and treatment was initiated. In this model adjusted for Age, Sex, Race, Diabetes status and Hypertension status, the initial eGFR is not different between the NON-RESPONDER and RESPONDER groups (p = 0.77). Over the follow-up time the eGFR for NON-RESPONDER (N = 108) is lower and declines over time (coefficient −0.007) compared to RESPONDER with a higher eGFR which increases over time (coefficient 0.004) (p < 0.001). No differences in the distribution of CKD stages between the two groups were noted (p = 0.21).

**Figure 3:**
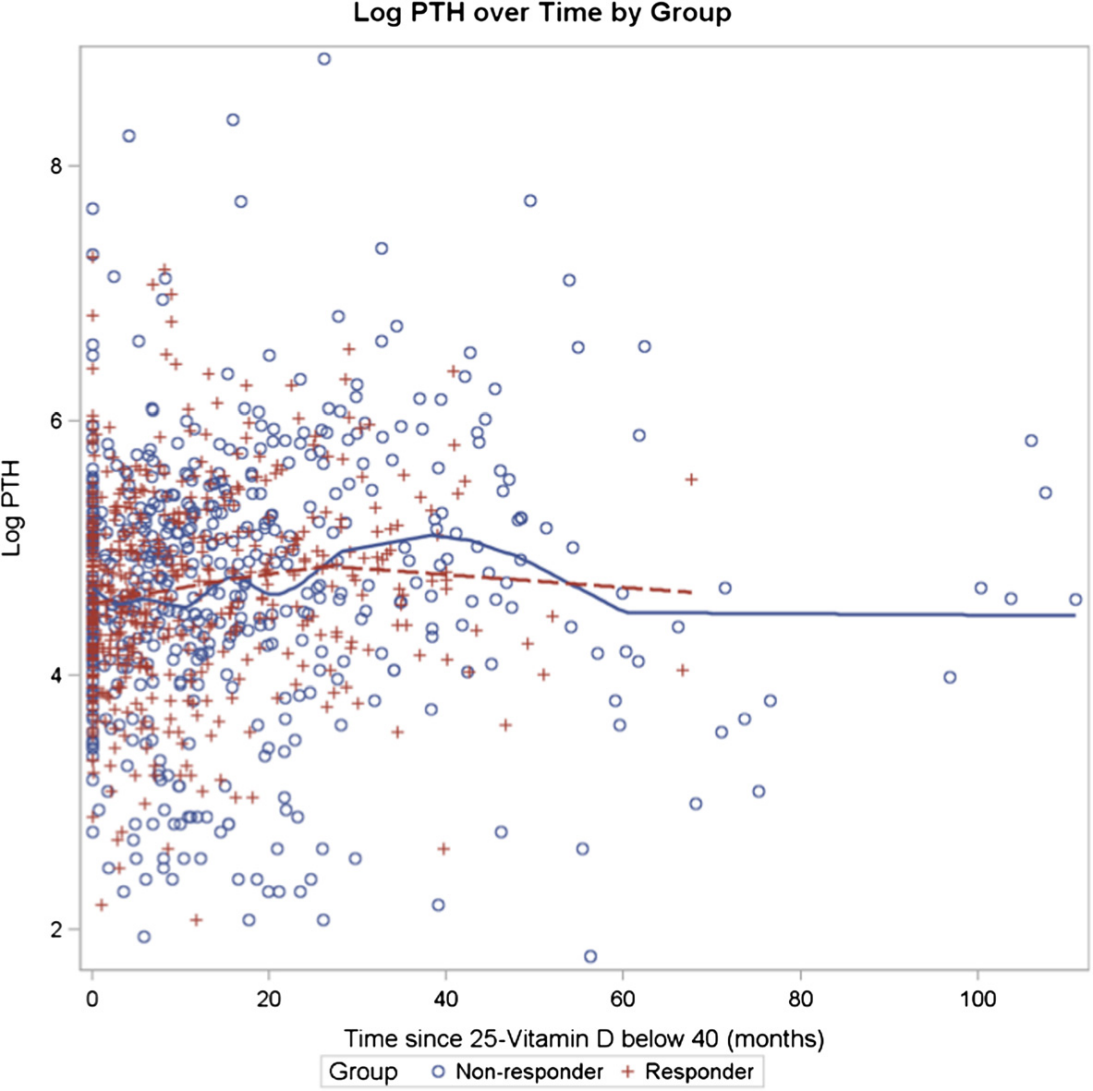
**Log PTH over time by group for CKD Stages 3–5.** Log PTH over Time by Group for CKD patients with eGFR < 60 ml/min/1.73 m^2^. N = 93 Non-responders and 119 Responders. There is no difference in PTH between the Non-Responders and Responders over time (p = NS).

Figure 3 depicts the relationship between PTH versus time in the RESPONDER and NON-RESPONDER groups for patients where the 25-OH vitamin D was below 40 ng/mL for CKD patients Stage 3–5 (N = 212) and treatment was initiated. There is no difference in PTH over time between the NON-RESPONDER and RESPONDER (p = NS). Similarly, there is no difference between 1, 25-OH vitamin D levels versus time and 25-OH vitamin D when the level was below 40 for CKD patients Stage 3–5 and treatment was initiated (figure not shown).

**Figure 4:**
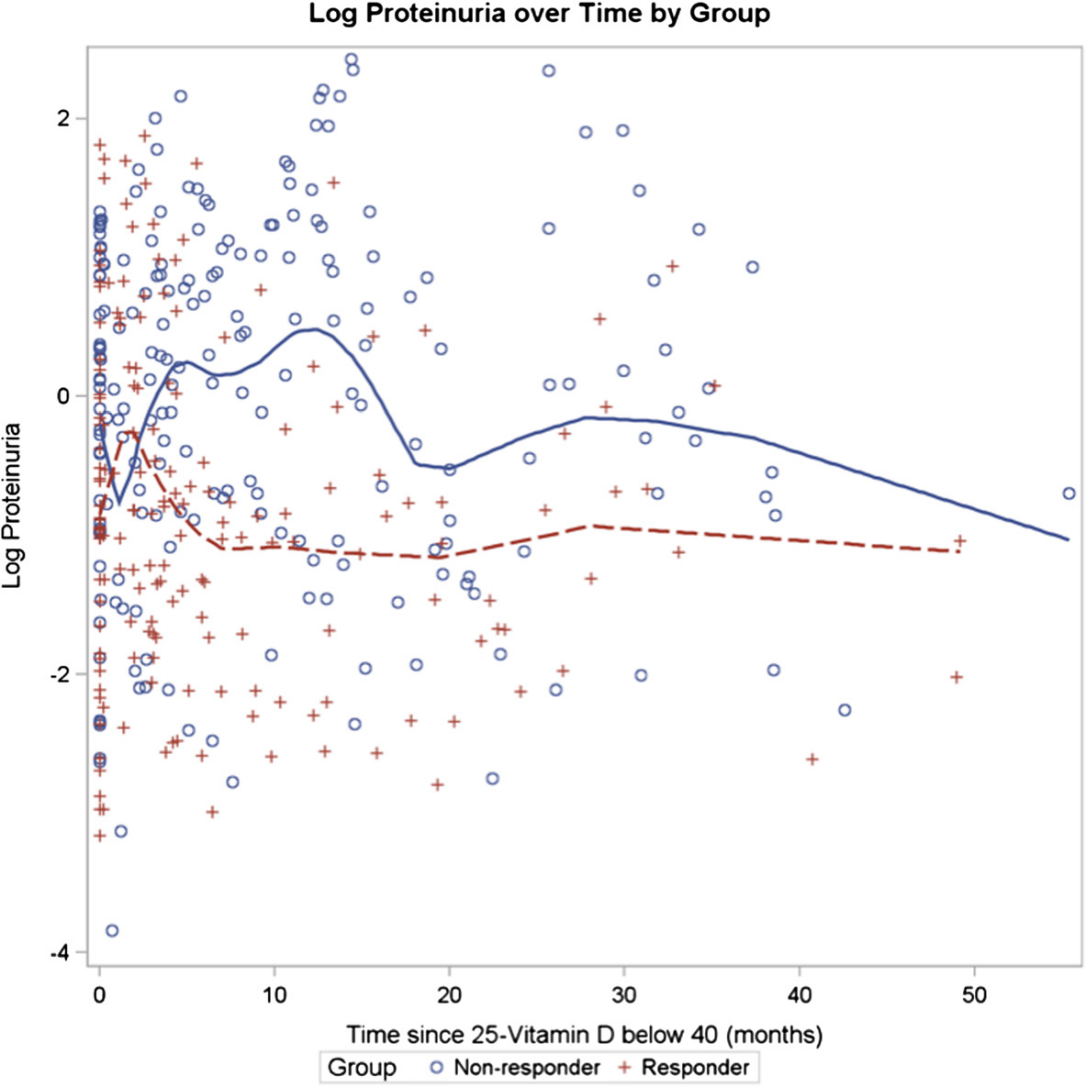
**Log Proteinuria over time by group for CKD patients with eGFR < 60 ml/min/1·73 m**^**2**^**.** Log Proteinuria over Time by Group for CKD patients with eGFR < 60 ml/min/1.73 m^2^. N = 69 Non-responders and 76 Responders. (p < 0.05). Proteinuria increases for 11 months in Non-responders. Proteinuria decreases for 21 months in Responders.

**Figure 5:**
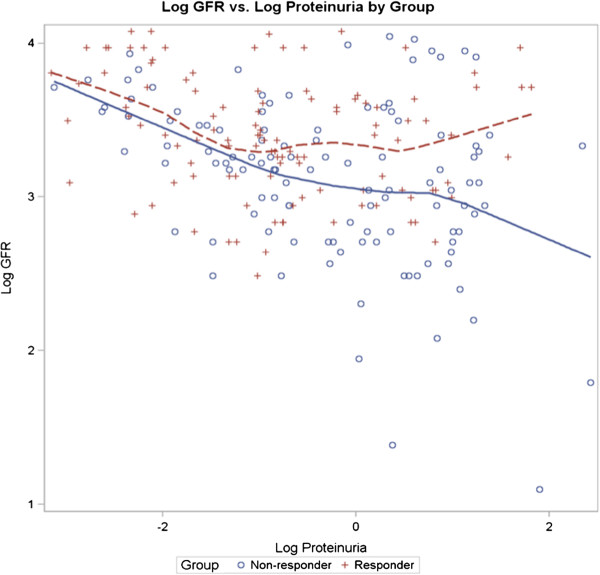
**Log eGFR vs. log proteinuria by group for CKD patients with eGFR < 60.** Log eGFR vs. Log Proteinuria by Group for CKD patients with eGFR < 60 ml/min/1.73 m^2^. N = 47 Non-responders and N = 45 Responders. For all levels of proteinuria, eGFR is lower in the Non-responders.

Figure 4 illustrates the Log Proteinuria over Time by Group for CKD patients with eGFR < 60 ml/min/1.73 m^2^. Proteinuria increases for 11 months in the NON-RESPONDER and decreases for 21 months in the RESPONDER. Figure 5 shows the Log eGFR vs. Log Proteinuria by Group for CKD patients with eGFR < 60 ml/min/1.73 m^2^. For all levels of proteinuria, the eGFR is lower in the NON-RESPONDER compared to the RESPONDER group.

## Discussion

Vitamin D insufficiency is highly prevalent in the CKD population. Across the United States prevalence ranges up to 70% [1]. This is consistent with our study in which 98% of all patients studied had vitamin D levels less than 40 ng/mL. Zisman suggested target levels of vitamin D inadequately suppress PTH in patients with CKD especially those with more advanced impairment in kidney function [11]. In the absence of published, definitive evidence based guidelines, our clinical practice guideline was to target vitamin D to levels of 40–60 ng/mL. This target was chosen based on a review of consensus opinions in the nephrology and endocrine communities [17,18]. We noted that this was exceedingly difficult to achieve in our population, which included both CKD and post-transplant patients.

One aim of our study was to demonstrate that cholecalciferol is a more potent vitamin D precursor therapy in CKD patients compared to ergocalciferol, which was used in previous studies [11]. What we uncovered is a new concept of vitamin D treatment resistance in CKD which we suggest is associated with a progressive decline in renal function over time. Our study has demonstrated that CKD patients treated with cholecalciferol, similar to ergocalciferol, experience treatment resistance in raising vitamin D levels to a pre-selected target level. Although multiple studies have suggested that cholecalciferol is more potent [19,20], we could not find evidence that the response to treatment with cholecalciferol is any better than with ergocalciferol. We did however, find two distinct groups in our population: 1) those who responded to treatment (RESPONDER) and 2) those who did not (NON-RESPONDER). Among all the patients, initial laboratory values which were unadjusted for follow-up for the NON-RESPONDER group were significant for a lower initial eGFR, higher PTH, lower albumin, higher phosphate, and lower 1, 25-OH vitamin D.

Similar to other studies with ergocalciferol, our population received a vigorous supplementation protocol with cholecalciferol. This regimen resulted in a significant increase in vitamin D levels only for a specific population: RESPONDER. This group has unique characteristics compared to those whose levels did not increase to the pre-selected target level of greater than 40 ng/mL. In the CKD Stage 3–5 group, unadjusted initial mean eGFR and 25-OH vitamin D levels were higher in the RESPONDER group. When adjusted for time for CKD patients with an eGFR < 60 ml/min/1.73 m^2^ the initial eGFR in RESPONDER and NON-RESPONDER groups is indistinguishable (Figure 2). The data suggests that the eGFR for the RESPONDER group stabilizes or increases over time, whereas the NON-RESPONDER group has a progressively worsening eGFR. This association however may represent the possibility that RESPONDER group overall had a better health status (i.e. higher albumin, lower phosphorus, higher initial eGFR). Adherence to the treatment regimen may possibly distinguish the RESPONDER and NON-RESPONER groups. However, compliance was assessed for all clinic patients on each visit and this effect is unlikely to be caused by non-adherence.

There are numerous hypotheses as to why one group might respond or not respond. Timing of administration or changes in intestinal absorption could account for a difference. In our study, transplant patients have a lower prevalence of NON-RESPONDER compared to the CKD population which may indicate that “healthy kidneys” play a role. Traditionally, calcidiol (25-OH vitamin D) levels are thought to be indicative of vitamin D body stores and correlate with nutritional status, whereas calcitriol (1, 25-OH vitamin D) levels are typically preserved despite the nutritional state [21-23].

It is possible that proteinuria with the loss of vitamin D binding protein may account for both the low levels of vitamin D observed and the vitamin D resistance in CKD [24-26]. Others have reported a favorable effect of vitamin D repletion on nephrotic range proteinuria [27,28]. Our data shows that response to treatment with cholecalciferol is associated with a lower initial level of proteinuria (Table 3) and a better preservation of eGFR (Figure 2). For any given level of proteinuria, the eGFR is lower in the NON-RESPONDER compared to the RESPONDER group (Figure 4). Our 3-way mixed model demonstrates that proteinuria modifies the time relationship between eGFR and the RESPONDER vs. NON-RESPONDER groups (p = 0.03), however it does not predict the change in eGFR over time. Proteinuria modifies, but does not account for, the vitamin D resistance.

Hormonal regulation and dysregulation are the hallmark features of CKD. The target of the hormonal dysregulation is centered on maintaining the serum calcium and phosphate concentrations in what we think is a normal target range well into the advanced stages of CKD. Reductions in 1,25-OH vitamin D are the single most prevalent abnormality characterizing CKD [29,30]. Concurrent with this reduction is the progressive elevation of FGF23, a hormone produced in the osteocyte of bone whose regulation has remained elusive [31,32]. PTH elevations are also progressive in CKD as a consequence of the reductions in 1,25-OH vitamin D over time. However, treatment with vitamin D also elevates FGF23 levels by virtue of a well described feedback loop [33]. High FGF23 levels have been shown to be a marker of poor patient outcome in both CKD and ESRD [34-36]. However, a recent animal study in a CKD model showed that targeted reduction in FGF23 resulted in increased mortality further complicating our understanding of this complex physiology [37]. In large data base studies of patients with CKD and ESRD, treatment with vitamin D has been shown to promote a survival advantage [38-42]. Calcitriol in high dose has been shown to promote vascular calcification in animal models of CKD [43-45] and a recent meta-analysis suggests that low-dose paricalcitol may do the same [46]. Thus the clinician in practice is faced with the clinical conundrum and safety concern of how much vitamin D to administer to patients with vitamin D insufficiency and to aim for what vitamin D target level in order to achieve the best clinical outcome. In our current understanding of this complex system, treatment with 25 vitamin D precursors and active vitamin D analogues is initiated to target PTH primarily to limit the progressive bone injury that occurs with elevated PTH levels. The PTH target is also a matter of much debate in the nephrology community [47].

The understanding of the interaction between active and inactive forms of vitamin D and PTH has improved immensely with the identification of FGF-23 [48], Klotho [49,50], and CYP27B1 [51]. Some have suggested that high doses of vitamin D supplementation similar to what was administered in our study induce the catabolic cytochrome P-450 enzyme CYP24A1 [52]. This may explain why high dose vitamin D supplementation and previous attempts of vitamin D repletion at lower dosage have failed [53]. Another potential mechanism which enhances vitamin D catabolism relates to the progressive elevation of the phosphaturic FGF23 noted in CKD. Both the administration of vitamin D and an elevated FGF23 in CKD have the potential to hyper-catabolize vitamin D in all forms. Therefore, an elevated FGF-23 may limit the response to a preselected vitamin D target level (i.e. vitamin D resistance) through this hypercatabolism mechanism.

Limitations of the study include the retrospective nature of the study design. Despite our best efforts to ascertain medication compliance, some patients may still have not taken their cholecalciferol. Seasonal differences were not investigated due to the long follow-up period of each patient. Cumulative doses of cholecalciferol given were not available. Weight, changes in weight, or the role of obesity in both groups was not ascertained. Given the low numbers of patients receiving home hemodialysis (3), outpatient center hemodialysis (23), and peritoneal dialysis (20) in our study, they were grouped together as renal replacement therapy and excluded from the study analysis. Conclusions could not adequately be drawn regarding these groups however the benefits of supplementation have been previously suggested in the PD population [6]. Information regarding malabsorption syndromes (such as by gastric bypass) of the patients in the study are unknown.

Multiple non-renal associations have been described between vitamin D: cardiovascular disease [54], immune function [55], asthma [56], cancer [57], and autoimmune diseases [58]. Low 25-OH vitamin D has also been suggested as a marker of mortality in the critical care setting [59]. Whether vitamin D becomes a marker of nutrition or of something more, monitoring of blood levels and targeted replacement therapy in the CKD population may give a hint of future CKD status. Our study is unable to identify suitable markers that could advance the state of knowledge and distinguish which patients will be in a future RESPONDER group and which will not.

## Conclusion

The clinical implications of a replete 25-OH vitamin D level remain unknown. Responders appear to stabilize or increase their eGFR over time, however the Non-Responders display a deterioration of eGFR over time. Proteinuria modifies the response to cholecalciferol initially but does not predict the change in eGFR over time. The mechanism of the treatment resistance (RESPONDER vs. NON-RESPONDER) is also unknown and could be caused by a change in vitamin D metabolism (decreased synthesis or increased catabolism). Both of these effects could be mediated by the progressive increase of FGF-23 levels observed in CKD. Further research is necessary to test this hypothesis and to identify predictive variables that could be used for prognostic guidance.

## Abbreviations

CKD: Chronic kidney disease; NKF: National kidney foundation; KDOQI: Kidney disease outcomes quality initiative; PTH: Parathyroid hormone; ICD-9: International classification of diseases ninth revision; eGFR: Estimated glomerular filtration rate; MDRD: Modified diet in renal disease; TXP: Transplant; RRT: Renal replacement therapy; FGF23: Fibroblast growth factor 23.

## Competing interests

The authors declare that they have no competing interests.

## Authors’ contributions

AP performed the literature search, study design, data collection, data analysis, data interpretation, and writing. HC performed data collection, data analysis, data interpretation, writing. LV participated in data collection, data analysis, and writing. LS performed literature search, study design, data analysis and interpretation, and writing. All authors had access to the data and a role in writing the manuscript. All authors read and approved the final manuscript.

## Pre-publication history

The pre-publication history for this paper can be accessed here:

http://www.biomedcentral.com/1471-2369/15/47/prepub
